# Should I eXtract Every Six dental trial (SIXES): study protocol for a randomized controlled trial

**DOI:** 10.1186/1745-6215-14-59

**Published:** 2013-02-27

**Authors:** Nicola Innes, Felicity Borrie, David Bearn, Dafydd Evans, Petra Rauchhaus, Steve McSwiggan, Lyndie Foster Page, Fiona Hogarth

**Affiliations:** 1 Dundee Dental Hospital and School, Park Place, University of Dundee, DD1 4HN, Dundee, UK; 2 Tayside Medical Sciences Centre, George Pirie Way, Ninewells Hospital, DD1 9SY, Dundee, UK; 3 Department of Oral Rehabilitation, Faculty of Dentistry, Sir John Walsh Institute, University of Otago, Dunedin, New Zealand

**Keywords:** First permanent molar, Compensating extraction, Orthodontics, Pediatric dentistry, Primary care, Randomized control trial, Dental, Oral health-related quality of life

## Abstract

**Background:**

Extraction of lower first permanent molars in children is common. There is uncertainty among clinicians as to whether a ‘compensating extraction’ (removal of the upper first permanent molar to prevent it over erupting) is necessary despite current guidelines recommending this. As a result, unnecessary dental extractions may be carried out or children may be failing to receive extractions required to achieve optimal long-term oral health. In addition, the decision to extract fewer or more teeth affects management options (local anesthetic injections alone, inhalation sedation or general anesthesia) needed to support the child with the surgical procedure(s).

The SIXES (Should I eXtract Every Six) dental trial investigates clinical effectiveness and quality of life for conventional treatment (following the guideline of compensation extraction of the upper first permanent molar) compared with the alternative intervention (removal of lower first permanent molars but no extraction of the upper).

**Methods/Design:**

This is a multicenter, two-arm parallel group randomized clinical trial. Allocation will be web-based randomization. Practitioners in primary and secondary care settings, reflecting the points of presentation and treatment of eligible patients, will recruit 400 children, aged 7 to 11 years requiring extraction of lower first permanent molars but who have upper first permanent molars of good prognosis. Baseline measures (prior to treatment) and outcome data (at one and five years, or when the patient reaches 14 years of age) will be assessed through study models and child/parent questionnaires.

The primary outcome measure is degree of tipping of the lower second permanent molar, (favorable outcome is tipping less than 15°).

The secondary outcomes are type of anesthetic/sedation used, residual spacing (between lower second premolar and second permanent molar), orthodontic treatment requirement, quality of life, and over-eruption in the intervention group. Assessors will be blinded where possible.

**Discussion:**

SIXES dental trial investigates whether compensating extraction of upper first permanent molars should be carried out following loss of lower first permanent molars. Currently dentists and orthodontists face a dilemma in clinical decision-making, relying on the lowest level of evidence - expert opinion. SIXES will provide evidence to support decision-making and inform practices and may result in reduced tooth extractions.

**Trial registration:**

Clinical Trials.gov Identifier: NCT01591265

## Background

### Introduction

The first permanent molars (FPMs) are the permanent teeth most commonly assessed as having a poor prognosis due to them being the permanent teeth most susceptible to dental caries in childhood [1,2] and the high prevalence of molar-incisor hypomineralization (MIH) [3,4]. It is accepted that while forced extraction of FPMs is rarely ideal, with appropriate timing and case selection, it can result in an acceptable occlusion for the child [5,6]. Preferably, all cases where planned loss of FPMs is anticipated should be managed jointly with an orthodontist, but this is not always possible. Treatment planning is complicated by the need to consider other factors alongside occlusal aspects such as the possible absence of other permanent teeth, the prognosis of the remaining molars, the anticipated cooperation of the child with any proposed restorative treatment, and socioeconomic factors. In addition, FPM teeth are the largest in the dentition, and their successful extraction places demands on both the child and the clinician.

To assist dentists in treatment planning, a national guidance document is available [7]. As well as advice on timing of extractions, this guidance recommends that in Class I and Class II cases, the extraction of a mandibular FPM should be compensated by the extraction of the opposing maxillary FPM. The rationale for compensating the extraction of a lower FPM is that an unopposed maxillary FPM may over-erupt and prevent mesial migration of the erupting lower second permanent molar. However, it has been suggested that there is little evidence to support this being a significant risk [8]. Furthermore, the extraction of an additional FPM places an increased burden on both the child and the dental team providing care for them, and may tip the balance of decision as to whether the extractions can be carried out under local anesthesia, towards favoring dental general anesthesia [9].

In summary, current clinical guidelines state that when a lower FPM is extracted, the upper FPM should also be extracted, yet this recommendation is based on little or no evidence. This randomized clinical trial will provide the evidence to answer this question: Is it necessary to extract the upper FPM routinely when extracting the lower FPM in the mixed dentition in order to prevent over-eruption of the upper FPM, which can cause occlusal problems?

Should this study find the procedure to be unnecessary, it will result in a reduction in the number of teeth extracted. The impact of the study would then be significant for children and their carers, as well as providers and purchasers of dental treatment.

### Rationale for the study

#### Do unopposed molar teeth over erupt?

There is good evidence that unopposed permanent molar teeth can over erupt. Kiliaridis [10] reported on 84 permanent molar teeth, which were known to have been unopposed for a minimum of 10 years, in 53 adults (mean age 65, range 40 to 89). While 18% of the molars showed no over-eruption (to visual examination of study models), 58% had over-erupted by up to 2 mm, while 24% had over-erupted in excess of 2 mm. There was no difference detected between maxillary and mandibular molars.

One cross-sectional study of 100 adults [11] with unopposed molar teeth, and a matched control group of 100 adults with opposing molars, reported over-eruption in 92% of unopposed molar cases. There was a mean value of 1.44 mm more over-eruption in the unopposed molar group than in the control group with opposing molars. This was less than 2 mm in 73% of cases. The extent of over-eruption was significantly greater in maxillary unopposed teeth than in mandibular unopposed teeth.

In a study of 91 adults who had molars which had been either partially or completely unopposed for a minimum of five years [12] partial occlusal contact did not inhibit overeruption but did increase the likelihood of tipping. Smith [13] looked at overeruption of lower second permanent molars in 42 patients following loss of maxillary second molars, comparing them with 42 matched control patients. Overeruption of the unopposed mandibular second molars was found to occur, but this was largely confined to the distal aspect (which was unopposed by the maxillary FPM), with a mean value of less than 1mm. Christou [14] and Compagnon [15] both reported overeruption of unopposed posterior teeth over time but, again, these studies involved adults.

#### Is potential overeruption of a maxillary first permanent molar a significant risk to the development of a healthy occlusion?

For patients within the age range for consideration for planned loss of FPMs, there are two possible complications of over-eruption of an unopposed maxillary FPM: 1) prevention of desired mesial movement of the mandibular second permanent molar and 2) the development of occlusal interferences, leading to temporo-mandibular joint dysfunction syndrome (TMDS).

#### Prevention of mesial movement of mandibular second molar

The Royal College of Surgeons of England (RCS) national guidance document [7] cites Holm [16] as the main data source for supporting compensating extractions following loss of lower FPMs. This paper is a conference report, reviewing 1,119 cases involving loss of one or more FPMs over a 10-year period at the Hamburg Public Orthodontic Institute. The review was carried out principally to assess the proportion of cases involving loss of FPMs, and the patterns of extraction. Although the poorest outcomes were found in cases of uncompensated extraction of lower FPMs, no data were presented to support this. In contrast, Mejare [8] reviewed 32 patients (mean age 18 years) who had loss of one or more FPMs in childhood (mean age 10 years) due to molar incisor hypomineralization (MIH). Of this sample, five patients had an uncompensated extraction of a lower FPM, and in none of these patients was over-eruption of the upper FPM noted. In addition, in a longitudinal study of 27 children who had one or more FPMs extracted due to MIH, Jalevick [17] reported no significant occlusal problems with the four children with uncompensated extractions of lower FPMs, and recommended against the need for compensating extractions. Patient satisfaction was also investigated in this study, and it was reported that of the five children assessed as having need of orthodontic correction of their occlusion, three of them declined treatment as they felt there was no need for it.

#### Development of occlusal interferences

Craddock, [18] in a cross-sectional study of 100 adult patients with at least one unopposed posterior tooth, and 100 control subjects, reported that 53% of the patients in the unopposed molar group had retruded contact position contacts or excursive interferences, compared with 12% of the controls. However, Agerberg [19] reported in a study of 140 teenagers and young adults that 89% of all subjects showed at least one occlusal interference, and that no correlation between the number of missing teeth and the number of interferences could be found. Heikinheimo [20], in a longitudinal study of 167 adolescents, found that none had a functionally optimal occlusion at 15 years of age, and that although around 30% of the sample had some tenderness to palpation of the muscles of mastication, none was found to have significant symptoms of TMDS.

## Trial Purpose

The purpose of this trial is to provide reliable evidence for clinicians as to whether compensating extraction of upper FPMs (as per current guidelines) should be carried out following loss of lower FPMs.

## Trial Objectives

The objective of this trial is to determine whether compensating extraction of upper FPMs following loss of lower FPMs in children is of benefit. The particular benefits being investigated are related to the resulting occlusion and oral health-related quality of life.

## Methods/Design

### Overview

**Figure 1:**
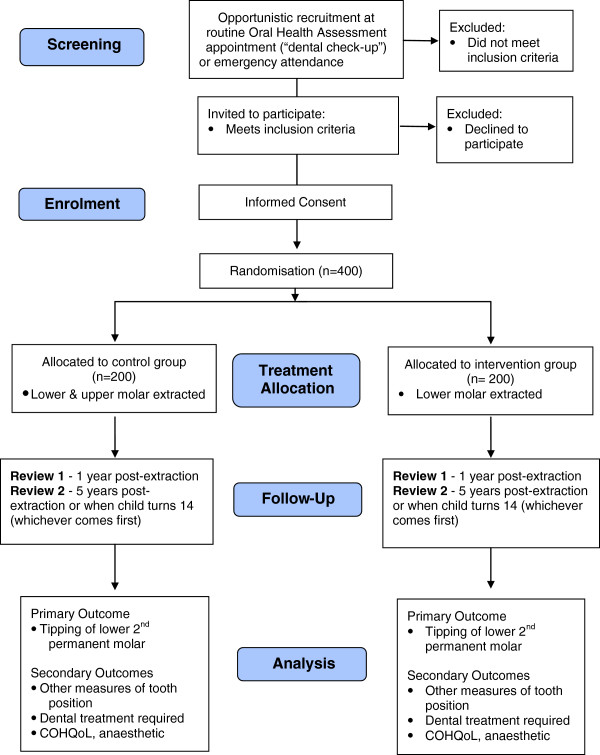
Screening, recruitment, randomization and participant follow-up schedule of the SIXES Trial.

**Figure 2:**
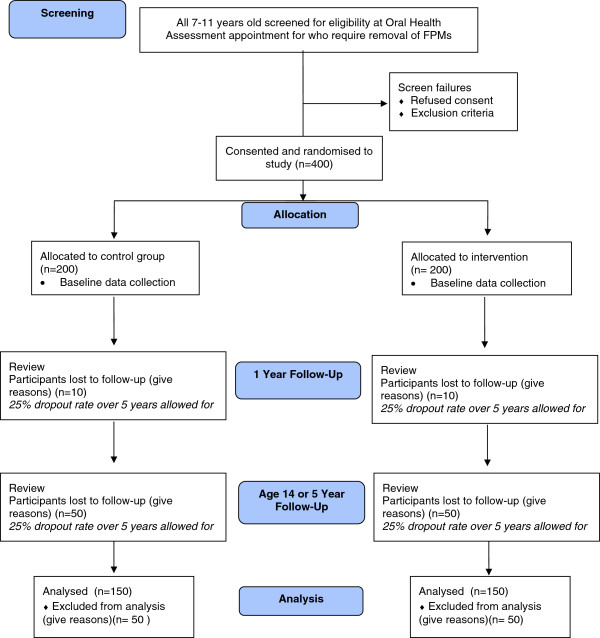
**SIXES trial flow diagram.** CONSORT [21] flow chart with projected numbers of participants throughout trial.

The SIXES Trial is a multicenter, two-arm parallel group randomized controlled trial using a superiority framework (see Figures 1 and 2). Measurements will be taken at one year after treatment and then again at either five years after treatment or when the patient reaches 14 years of age, whichever occurs first. The trial will be set in both primary and secondary care settings, reflecting the points of presentation and treatment of eligible patients. Figure 1 shows details of screening, recruitment, randomization and participant follow-up schedule and Figure 2 is a trial CONSORT type flow diagram [21] with projected numbers of participants throughout the trial.

### Basis for the study design

This study will recruit patients from both secondary and primary care settings and include general dental practitioners, pediatric dentists and orthodontists in order to attract a representative sample of patients (by wide net). This study design is based around routine care as practitioners use both the current guidelines and the proposed intervention in their treatment of patients. It aims to assess these practices relative to one another and, as such is a low-risk study assessing current practice.

Study recruitment is taking place over a 24-month period commencing in July 2012, with the coordinating site being the Dundee Dental Hospital Orthodontic and Paediatric Dentistry Departments. Other sites will be recruited to the trial.

Dentists are considered eligible to participate if they treat child patients and are willing to undergo good clinical practice (GCP)/research governance framework (RGF) training. Tailored training relevant to dental studies involving children for study GCP and RGF is being delivered by Tayside medical Science Centre (TASC).

Prior to study recruitment at any individual site, there will be a site initiation visit and site staff will receive training on the study design, methodology, clinical intervention and on maintaining trial documentation, including the CRFs, questionnaires and investigator site file.

### Participant inclusion criteria

Age: 7 to 11 years

Dental History: Able to cooperate with dental treatment Regular attender or considered likely to return for follow-up

Social History: Child and carer able to understand study documentation and give consent to participate in study

Dental condition: One or two lower FPMs requiring extraction Upper FPMs are sound or restorable/restored with good long term prognosis (that is, has or requires a single surface restoration with caries less than half-way into dentine, restoration with a simple restoration)Confirmed presence of all second premolars and all second molars

### Participant exclusion criteria

Medical history: Medical contraindication to dental extractions

Dental condition: Poor prognosis of premolars or permanent second molar teethAll four upper incisors in crossbite Poor prognosis of upper FPM Confirmed absence of one or more second premolars and second molarsDeclines to have impressions taken.

### Interventions

The patient will continue to have all other treatment planned and carried out as normal. The only difference in treatment will be related to the FPM extractions (one side only per patient will be entered into the trial). For patients allocated to the control arm, both the upper FPM and lower FPM will be extracted. For patients in the intervention arm, only the lower FPM will be extracted.

Only one side of the mouth will have data collected for the study. For patients who require extraction of lower FPMs on both sides of the mouth, the decision of which side to include in the study will be taken through randomization.

The control group will have the normal standard intervention according to the current standard guidelines, and will receive routine patient care with the normal practice of compensation extraction, while the intervention group will have removal of their lower FPMs but no compensation extraction of the upper FPM will be carried out.

### Training in intervention

As the proposed intervention is removing a tooth, which is standard clinical practice, no specific training on that procedure is required.

### Participant timeline

Patients will be identified by clinicians participating in the trial from either the patients’ own dentist or orthodontist; or a secondary care orthodontic or pediatric department.

Patients who are found, during routine dental examination or assessment, to require extraction of a lower FPM will be assessed by their dentist or orthodontist for meeting the inclusion criteria. Patients who meet the inclusion criteria will have the study discussed with them and their parents/guardians by their clinician. A participant information leaflet will be given. Patients will be given a further appointment for the extractions, at least 24 hours after receiving the information and at this time, the study will be discussed again. However if treatment is to be carried out at the same appointment (for example, where patients are in pain), there will be time allowed for the patient and parent/carer to read the documentation and ask any questions to ensure they have sufficient time and information for consent to be informed. Consent/assent processes will be carried out and then randomization undertaken. Extraction(s) will be carried out as per randomization and the remaining treatment plan.

The patient will complete the first questionnaire and have upper and lower impressions taken for study models to be cast. When the patient returns for 1-year follow-up, they will complete the Year 1 Follow-up Questionnaire and have upper and lower impressions taken for study models to be cast. When the patient returns for final follow-up (at five years or when the patient has reached 14 years of age), they will complete the Final Follow-up Questionnaire and have upper and lower impressions taken for study models to be cast.

### Target sample size

The sample size calculation is based on the primary outcome of tipping of the lower molar tooth. There are no published data on which to base the calculation, therefore local audit data were used. These data indicated that tipping of the molar (a dichotomous outcome of either less than or more than 15 degrees tipping in the vertical direction) lies somewhere between 50:50 and 60:40 for good to poor outcome in a representative group. A finding of a difference in this proportion of 10 percent between the two groups, for example proportions of 50:50 and 40:60, in the two comparison groups would be deemed to be clinically significant.

Sample size calculation for a power of 0.8 and an alpha of 0.05 indicated a sample of 124 in each group if the true population proportion is 60:40, or 171 if the proportion is 50:50. It was therefore decided to set the required sample size at 150 in each group, requiring 300 in total. Allowing for a drop-out rate of 25% our target sample size is therefore 400.

### Dentist recruitment and retention strategies

Recruitment of dentists has begun and will continue to take place in the settings from which participants will be recruited:

1. Secondary care (NHS Tayside): at Dundee Dental Hospital and School, in the Orthodontic and Paediatric Department, all clinicians who see children for routine check-up appointments or non-urgent secondary care assessments, have been invited to participate in the recruitment and follow-up of children in the study. Good clinical practice (GCP) trained staff will be able to consent patients.

2. Primary care practices (including NHS Tayside practices) and orthodontic practices: in Scotland, General Dental Practitioners (GDPs) who are part of the SDPBRN Rapid Evaluation Network and dentists who have participated in other trials will be invited to participate.

**Figure 3:**
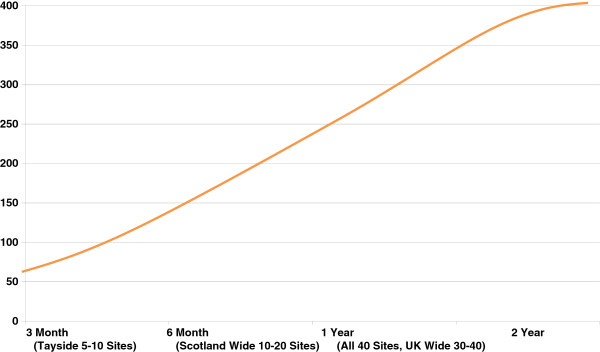
Projected recruitment and study site enrollment.

It is expected that up to 40 sites will be recruited to participate in this trial. Sites will be initiated in batches of 5 to 10 sites per recruitment period. A batch of sites in an area will be started and recruitment targets reviewed before a new set of sites is initiated (See Figure 3).

To help with retaining dentists in the study, the following strategies are being employed:

1. The trial manager will maintain contact with the practitioners, manage any non-clinical queries and refer clinical problems to the dental staff.

2. Continuous Professional Development points will be made available for attendance at any meetings.

3. Newsletters will be issued during the 2 years of patient recruitment, then at 3 years and 4 years (to mark the 1 year review) and at the final 6 and 7 year time points to update dentists on trial progress and help to maintain engagement with the study.

4. A final report will be issued to all of the participating dentists.

### Participant recruitment and retention

Patients who meet the inclusion criteria will receive an invitation to take part in the trial will be identified during attendance at appointments with clinicians participating in the trial.

It is anticipated that the majority of patients will be attending for a six monthly routine check-up appointment (primary care GDP or community dentist) or a non-urgent secondary care assessment (practice-based specialist orthodontist, hospital-based orthodontic service and pediatric department).

Patients attending for routine check-up appointments or non-urgent assessments, who meet the inclusion criteria, will be assessed by the dentist for eligibility, and asked whether they wish to participate when they return for treatment at their next appointment. The patients and parents will be given information on the study to take home with them. Consent will be taken at the subsequent appointment by a dentist participating in the trial as a principal investigator (PI). Randomization will be carried out prior to this appointment and treatment.

For children who attend for urgent appointments, presenting in pain, the patients and their parents will also be approached at the time of their appointment about whether they wish to participate in the study. Because there is the need to alleviate the child’s symptoms as soon as possible, usually involving extraction of the tooth, there is no scope for 24 hours to reflect on the trial information. For this reason some patients will be invited to participate at their assessment appointment. Consent and randomization will still follow best practice, and parents and patients will still be given time to look at the patient information sheets and consider the study. The intervention in this study (extraction of a single tooth compared with extraction of two teeth) still allows the patient to return for conventional treatment (extraction of the upper FPM) should they be allocated to the intervention arm but change their mind about participation at a later date.

On enrollment to the SIXES trial, parents and patients will be given ‘calling cards’ saying that they are participating in the study, with details of their dentists and who to contact regarding the study.

### Participant allocation

Sequence generation and randomization will be via a centrally controlled web-based GCP compliant randomization system, run by Tayside Clinical Trials Unit (TCTU). Randomization will be stratified by site. To ensure balanced assignment across critical variables, a minimization algorithm will be employed, using baseline age.

Participants will receive the intervention or the control as per randomization schedule.

Stratification will be by age, into two groups: 7 and 8 years and 9, 10, and 11 years.

### Blinding

Due to the nature of the proposed intervention it is not possible to blind staff, the children or their carers to the group once it has been allocated.

### Data collection, management and analysis

#### Primary outcomes

The primary outcome measure is extent of tipping of the lower second permanent molar, with a favorable outcome being a degree of tipping less than 15° and unfavorable outcome being greater than 15°.

Participants will be allocated into ‘favorable’ or ‘unfavorable’ outcome groups when comparing study models.

#### Secondary outcomes

The secondary outcomes for the study are:

1. position of the upper FPMs with regard to over-eruption^a^;

2. residual spacing between the lower second permanent molar and the lower second premolar^a^;

3. American Board of Orthodontics (ABO) scores^a^;

4. type of anesthetic used during procedures^b^;

5. dental or orthodontic treatment carried out during the follow-up period^b^ ;

6. Child and Parent Oral Health-Related Quality of Life (OHRQoL) scores (child and parent questionnaires).

#### Data collection

Data will be collected at three points (see Table 1).

**Table 1:** SIXES study visit schedule

	**Baseline (T0)**	**1-year follow-up (T1)**	**5-year follow-up (T2)**
Check inclusion/exclusion criteria	**X**		
Complete child’s assent and parental consent forms	**X**		
Randomization via website	**X**		
**Case report form to be completed by dentist**			
Baseline demographic information	**X**		
Dental history	**X**		
Record intervention or control arm	**X**		
Record side of treatment (right or left)	**X**		
Record stage of development of lower second molar	**X**		
Record type of anesthetic/sedation used	**X**		
Record dental treatment to remaining upper first permanent molars	**X**	**X**	**X**
Record orthodontic treatment since intervention		**X**	**X**
**Questionnaires**			
Child’s oral health quality of life questionnaire	**X**	**X**	**X**
Parental oral health quality of life questionnaire	**X**	**X**	**X**
**Other clinical records**			
Dental impressions and occlusal registration	**X**	**X**	**X**

At time of extraction (T0):

1. Dental impressions and a record of occlusal registration will be taken for study models

2. The dentists will complete a case report form (CRF) (patient details, which arm randomized to, which side treatment was allocated to, what kind of anesthetic was used - local anesthesia (LA)/inhalation sedation (IHS)/general anesthesia (GA)/other type of sedation)

3. Children’s Oral Health-Related Quality of Life (OHRQoL) questionnaire

4. Parental OHRQoL questionnaire

After 1 year (T1):

1. Dental impressions and a record of occlusal registration will be taken for study models

2. Record of any dental treatment (to remaining upper FPMs) or orthodontic treatment provided since initial intervention

3. Children’s OHRQoL questionnaire

4. Parental OHRQoL questionnaire

After 5 years or when child turns 14 (whichever is first) (T2):

1. Dental impressions and a record of occlusal registration will be taken for study models

2. Dentists will record any dental treatment (to remaining upper FPMs) or orthodontic treatment provided since initial intervention

3. Children’s OHRQoL questionnaire

4. Parental OHRQoL questionnaire

The CRFs and questionnaires will all be marked with the patient’s unique study ID number which will be linked to the enrollment log for the trial.

Study models will be 3D scanned to measure occlusal changes. All patients’ lower study models will be assessed with regard to the tipping of the lower second permanent molar relative to the occlusal plane. The scanning software will be able to create an occlusal plane from cusp tips in order to calculate the degree of molar tipping. Not all patients will have an erupted lower second permanent molar at the earlier time points in the study.

Only the groups of patients who do not have the upper FPMs extracted will require their upper study models to be assessed for FPM over-eruption. Each patient’s upper models will be superimposed on the palatal rugae and any movement in the *x, y* and *z* plane of the upper FPMs will be recorded.

In addition, the 3D scans of the models will be used to give ABO occlusal index scores and to assess and measure any residual spacing between the lower second permanent molar and the lower second premolar.

#### Quality of life measures - child

Child and adolescent OHRQoL measures document the functional and psychosocial outcomes of oral disorders. It is generally accepted that these measures are as essential as clinical indicators when assessing the oral health of individuals or populations, making clinical decisions and evaluating dental interventions, services and programs [22]. Finding a tool for measuring oral health-related quality of life in children is complicated by the rapid changes seen as children grow [22,23]. This study includes children as they age from 7 years to 14 years. The Child Perceptions Questionnaire (CPQ), which has been validated for children 8 to 10 years old [24], and the short-form version for children 11 to 14 years old will be used [25,26]. It has been found to have acceptable internal consistency, reproducibility, criteria and construct validity when used in a dental clinic/practice population in the United Kingdom [27,28].

These measures of oral health-related quality of life will be taken at baseline (T0), after one year (T1) and after 5 years or when the child turns 14, whichever comes first (T2). Comparisons will be between groups at each time point. Consideration will be given to analyzing data between time points depending on the quality of the data gathered and the outcomes of research currently underway by other groups which will be published by the end of this study’s data collection.

#### Quality of life measures - parents

Parents, will also complete an OHRQoL questionnaire consisting of the one global rating and questions from the short-form Parental Caregivers Perceptions Questionnaire (SFPCPQ), which is the parent version of the CPQ 11–14. These measures of oral health-related quality of life will be taken at baseline (T0), after one year (T1) and after 5 years or when the child turns 14, whichever comes first (T2). Comparisons will be between groups at each time point. As with the children’s OHRQoL measures, consideration will be given to analyzing data between time points depending on the quality of the data gathered and the outcomes of research currently underway by other groups which will be published by the end of this study’s data collection.

#### Type of anesthetic

Data will be collected through the CRF on the type of anesthetic used during the extraction of FPMs (for example, whether any form of sedation or general anesthetic was used for each of the teeth extracted).

#### Orthodontic/dental review

The dentist will complete a questionnaire regarding dental and orthodontic treatment at 1 year follow-up and final follow-up to find out whether:

1. the upper FPM is still present (for participants in the intervention group);

2. the upper FPM has required dental treatment and if so, details of the treatment (for participants in the intervention group);

3. the patient had, or are they currently undergoing, any orthodontic treatment? (If yes, the type of treatment);

4. they have experienced any symptoms of temporo-mandibular joint dysfunction syndrome (jaw pain).

#### Data management and statistical methods

Anonymized cast models will be 3D scanned and process data analyzed to record the type and extent of dental treatment (including the study interventions) that the subjects have received during the period of the study. This will be entered into a GCP compliant data management system Open Clinica (https://www.OpenClinica.com), which can be exported to SPSS (Statistical Package for Social Sciences, Inc., Chicago, IL, USA) v.18 for statistical analysis. Patient satisfaction questionnaires will be entered into Open Clinica and exported to SPSS for descriptive statistical analysis.

COHQoL data will be entered into Open Clinica and exported to SPSS for quantitative analysis of differences in overall and domain scores at the three time points using Friedman test for repeated measures

For between group comparisons, data will be checked for normality of distribution and any evidence of skewness. Descriptive statistics will be prepared. Appropriate parametric or non-parametric analysis allowing for any clustering effect will be undertaken to determine statistically significant differences in the outcomes listed above between the two study groups including chi-squared and *t*-tests, if normally distributed.

The proposed analyses will investigate comparisons between the intervention group, where patients have not had compensatory removal of FPMs, and the control group where patients have had this done. Specifically, we are looking for clinically significant improvement in:

•position of second permanent molars (if erupted);

•degree of tipping of the lower second permanent molars;

•spacing between lower second premolar and second permanent molars;

•position/over-eruption of upper FPM in the intervention group;

•ABO scores;

•dental/orthodontic treatment required during the study period;

•child OHQoL scores;

•use of anesthetics.

Missing data will be analyzed by multiple imputation up to the patient’s last recorded visit.

Interim analyses will not be carried out for this study as it will not be possible to measure the primary outcome until recordings are taken at 5-year follow-up, by which time patient recruitment will be complete.

### Trial management and monitoring

#### Trial monitoring

The TASC standard operating procedure (SOP) on adverse event (AE) recording will be adhered to for reporting of harms in this study.

This study is low risk and no adverse events are expected from this one-off intervention, which consists of a standard dental procedure (extraction of a tooth). The proposed intervention is less invasive than the standard procedure (and actually forms a part of it). Both the standard procedure and, therefore, the proposed intervention are considered unlikely to result in serious adverse events (SAEs) or suspected unexpected serious adverse reactions (SuSARS). Any participants reporting AEs would discuss this with clinic staff or may contact the trial team directly. All relevant AEs and SAEs will be recorded from the time a participant consents to join the study until day 10 post-initial treatment.

Hospital admissions, changes in concomitant medications, or other health problems will not be reported as AEs or SAEs unless there is direct evidence that the AE has been caused by the intervention. However, should they occur, they will be recorded in keeping with normal practice.

While it is anticipated that the incidents of SAEs and reactions to the treatments will be rare, there are a number of common, and well-understood consequences of the use of dental anesthetics (local anesthetic (LA), inhalation sedation (IHS), general anesthetic (GA), sedation) and extractions. A listing of the common and well-understood consequences of treatment, less common side effects, and rare events can be found in Table 2.

**Table 2:** Common and well-understood consequences of treatment

**Intervention**	**Adverse events**		
	**Common and well-understood consequences of treatment**	**Less common and unpleasant side-effects**	**Rare events**
**Extraction of tooth**	• pain around site	• early and delayed post extraction bleeding	• temporo-mandibular joint pain
	• swelling	• infection of socket	• fracture of mandible
	• loss of space for developing dentition	• fracture of tooth and surgical procedure to remove remaining portion	• oral-antral communication
**Fillings in teeth and crowns on teeth**	• occlusal discomfort	• pain, pulpitis	• trauma to soft tissues
		• localized reaction to bonding agents or filling materials	
	• damage to adjacent teeth	• dental abscess	
	• caries progression	• facial swelling	
**Local anesthetic injections**	• pain at site of injection (during or immediately following injection)	• self-inflicted trauma to soft tissues	• trismus
			• prolonged altered sensation
			• swelling
			• hematoma
			• allergic reaction
**Inhalation sedation**	• dizziness	• nausea	• loss of consciousness
		• headache	
**General anesthesia**	•nausea and vomiting	• reaction to anesthetic agent	• death
	• drowsiness	• sore throat or nose bleed (depending on type of intubation)	
	• shivering and feeling cold		

#### Trial management and oversight arrangements

The cosponsors of this trial are the University of Dundee and NHS Tayside.

The trial will be coordinated by the Trial Management Group, (TMG) consisting of the chief investigators, investigators, representative(s) of site PIs, TCTU senior clinical trial manager and a statistician. This group will meet regularly throughout the trial.

A TCTU trial manager will oversee and coordinate the study and will be accountable to the chief investigator. The PI at each site will be responsible for checking the CRFs for completeness, plausibility and consistency. Any queries will be resolved by the investigator or delegated member of the trial team.

A delegation log will be prepared for each site, detailing the responsibilities of each member of staff working on the trial.

The Central Trials Office, TCTU, will provide support to the study team. The office will be responsible for randomization, collection of data in collaboration with the trial manager, data processing and analysis. Publication and dissemination of the study results will be coordinated by the chief investigators and the TCTU, who have had an advisory role in this trial.

A Trial Steering Committee has been established to oversee the conduct and progress of the trial. This comprises: the co-chief investigators, co-investigators, a representative from Tayside Medical Sciences Centre, a practitioner representative and a patient representative.

An independent Data Monitoring Committee will not be necessary for this trial as the study is low risk.

Principal investigators and institutions involved in the study will permit trial-related monitoring, audits, Research Ethics Committee (REC) review, and regulatory inspection(s). In the event of an audit, the investigator agrees to allow the sponsor, representatives of the sponsor or regulatory authorities direct access to all study records and source documentation.

This study has been reviewed by the University of Dundee sponsorship review and is considered low risk, however to ensure the study is run to GCP standards the site will be regularly monitored by the Co-Chief Investigator, Felicity Borrie, or a representative of TASC as agreed by the sponsor.

#### Investigator responsibilities

The principal investigators are responsible for the overall conduct of the study at their site and compliance with the protocol and any protocol amendments. In accordance with the principles of GCP, the following areas listed in this section are also the responsibility of the investigator. Responsibilities may be delegated to an appropriate member of study site staff, which in this case are senior clinical dental staff who have been nominated as co-investigators. Delegated tasks must be documented on a delegation log and signed by all those named on the list.

#### Emergency code-breaking procedure

Emergency code-breaking in this randomized control trial is not applicable because the pattern of tooth extraction is visible.

The proposed intervention is unlikely to result in SAEs or SuSARS. We do not expect to report hospital admissions, changes in concomitant medications or other health problems as AEs or SAEs unless there is direct evidence that the AE has been caused by the intervention. However, should they occur, they will be recorded in keeping with normal practice.

### Ethical consideration

#### Research ethics approval

The study will be conducted in accordance with the principles of GCP. Approval will be obtained from the appropriate REC and local National Health Service Research and Development (R&D) approval will be obtained prior to commencement of the study at any site.

#### Protocol amendments

Any changes in research activity, except those necessary to remove an apparent, immediate hazard to the participant, will be reviewed and approved by one of the co-chief investigators. Amendments to the protocol will be submitted in writing to the appropriate REC and local R&D for approval prior to participants being enrolled into an amended protocol.

In the event that an investigator needed to deviate from the protocol, the nature of and reasons for the deviation will be recorded in the CRF. If this necessitates a subsequent protocol amendment, this will be submitted to the REC and local R&D for review and approval if appropriate.

#### Consent and assent

Where patients attend their own primary care dentist, these dentists will act as principal investigators and will obtain consent from the patients. Patients attending the Dundee Dental Hospital and receiving treatment there will give consent to one of the trial team wherever possible. If this is not possible, a suitably trained dentist will obtain consent. Regardless of location, consent of the patients will be performed by dentists who will be conversant with the study design and protocol, and be GCP trained in line with the Research Governance Framework (RGF).

The parent(s)/legal guardian(s) of all children in the study will provide written informed consent before any study procedures are carried out and a participant information sheet will be provided to facilitate this process. Where possible, and with the agreement of the parent(s)/legal guardian(s), participating children will also be asked to provide written or oral assent. Those not competent in English will be invited to bring an interpreter with them to the subsequent treatment appointment or to request an NHS interpreter, where this service is available.

As part of the consent process parent(s)/legal guardian(s) must agree to researchers and regulatory representatives having access to their medical records for monitoring and audit purposes. Parent(s)/legal guardian(s) may withdraw their consent to participate at any time during the study.

The investigator or delegated member of the trial team and the child should sign and date the Assent form whereas the parent and investigator should sign the Informed Consent form(s) to confirm that consent has been obtained. The participant should then receive a copy of this document and a copy should be filed in the investigator site file (ISF).

#### Withdrawal procedures

Parent(s)/legal guardian(s) will be informed that they have the right to withdraw their child from the study at any time. The right to refuse to participate without reasons will be respected. After the participant has entered the study the clinician remains free to give alternative treatment to that specified in the protocol at any stage if he/she feels that it is in the participant’s best interest, but reasons for doing so will be recorded. In these cases the participants remain within the study for the purposes of follow-up and data analysis. All participants will be free to withdraw at any time from the study without giving reasons and without prejudicing further treatment.

Due to the extended follow-up period for this trial (5 years), some children may be lost to follow-up. If a patient withdraws due to geographical reasons or because he or she is receiving treatment elsewhere, it may be feasible to send the review questionnaire to that patient, as we will attempt to track all patients using their CHI number by contacting their GP or GDP. Attempts will be made to continue data collection from the patient through the new GDP.

If the level of withdrawn participants is high it may be necessary to extend the recruitment period.

For participants who withdraw and do not wish to receive study related interventions, then no further contact will be made by the study team.

The study team can not foresee any incidences where a participant would be withdrawn for safety reasons as this is considered to be a one-off low risk intervention and involves routine dental practice of a common procedure (that is, dental extraction).

#### Confidentiality

All evaluation forms, reports, and other records will be identified in a manner designed to maintain participant confidentiality. All records will be kept in a secure storage area with limited access. Clinical information will not be released without the written permission of the participant, except as necessary for monitoring and auditing by the sponsor, its designee, regulatory authorities, or the REC. The Investigator and study site staff involved with this study will not disclose or use for any purpose other than performance of the study, any data, record, or other unpublished, confidential information disclosed to those individuals for the purpose of the study. Prior written agreement from the sponsor or its designee would be obtained for the disclosure of any said confidential information to other parties.

All investigators and study site staff involved with this study will comply with the requirements of the Data Protection Act 1998 with regard to the collection, storage, processing and disclosure of personal information and will uphold the Act’s core principles. Access to collated participant data will be restricted to those clinicians treating the participants. Computers used to collate the data will have limited access measures via user names and passwords. Published results will not contain any personal data that could allow identification of individual participants.

#### Post-trial care

All study documentation will be retained for at least 5 years post final data lock.

The end of study is defined as the last participant’s last visit. The end of the study will be reported to the REC within 90 days, or 15 days if the study is terminated prematurely. The investigators will inform participants and ensure that they have attended for a follow-up appointment (normally as part of their routine dental recall appointment). Where participants have failed to attend they will be contacted to arrange a follow-up appointment. A summary report of the study will be provided to the REC within 1 year of the end of the study.

### Dissemination of results and publication policy

The results of the trial will be presented at Orthodontic and Paediatric Dentistry National and International Conferences. The results will be used to inform National Guidelines, be published in peer-reviewed journals and inform teaching in Dundee University. All patients recruited into the trial will be given a summary of the trial findings after the final report is prepared.

Ownership of the data arising from this study resides with the study team. On completion of the study, the study data will be analyzed, tabulated and a clinical study report will be prepared.

The International Committee of Medical Journal Editors recommendation on Authorship and Contributorship will be adhered to when presenting papers for publication or acknowledging public responsibility for appropriate portions of the content.

### Trial status

SIXES Dental Trial is open for recruitment of patients with complete enrolment (n = 400) being projected as July 2014.

## Endnotes

^a^Outcomes 1, 2 and 3 will be measured on study models of the teeth, cast from impressions taken at baseline and after 1 year and 5 years or when the participant reaches 14 years of age, whichever is first.

^b^Outcomes 4 and 5 will be recorded by dentists on the Case Report Form.

## Abbreviations

ABO: American Board of Orthodontics; AE: adverse event; CI: chief investigator; CLRN: Clinical Research Network; CPQ: Child Perceptions Questionnaire; CRF: case report form;DDH: Dundee Dental Hospital; DMEC: Data Monitoring & Ethics Committee; FPM: first permanent molars; GA: general anesthetic; GCP: good clinical practice; GDP: general dental practitioner; IHS: inhalation sedation; ISF: investigator site file; LA: local anesthetic; OHRQoL: oral health-related quality of life; PCP-Q: Parental Caregivers Perceptions Questionnaire; PI: principal investigator (at each site); QoL: quality of life; R&D: research and development; RCS Eng: Royal College of Surgeons of England; RCT: randomized controlled trial; RGF: research governance framework; REC: research ethics committee; SAE: serious adverse event; SOP: standard operating procedure; SUSAR: suspected unexpected serious adverse reaction; TASC: Tayside medical Science Centre; TCTU: Tayside Clinical Trials Unit; TSC: Trial Steering Committee; TMDS: temporo-mandibular joint dysfunction syndrome.

## Competing interests

Although FB and DB are members of the British Orthodontic Society, there are no conflicts between this membership and their roles in participating in the trial, management of the data or publication of the trial findings. The authors declare that they have no competing interests.

## Authors' contributions

NI, FB, DE, DB conceived of the study and contributed to its design. NI, FB, DE, DB, SM, FH were involved in writing the study protocol/manuscript. PD or PR performed the statistical analysis and drafted the statistical section. NI, FB revised the manuscript critically for publication. All authors read and approved the final manuscript.
